# Voltage-gated sodium channel, Na_v_ 1.8, regulates calcium dynamics in the dorsal root ganglion induced by cold nociceptive stimulus

**DOI:** 10.1371/journal.pone.0357395

**Published:** 2026-09-18

**Authors:** Moushumi Rani Dey, Baolin Li, John Heddleston, Eitaro Aihara

**Affiliations:** 1 Biotechnology Discovery Research, Lilly Research Laboratories, Eli Lilly and Company, Indianapolis, Indiana, United States of America; 2 Neuroscience Discovery, Lilly Research Laboratories, Eli Lilly and Company, Indianapolis, Indiana, United states of America; Cinvestav-IPN, MEXICO

## Abstract

Nociceptive pain responses are mediated by neurons in the dorsal root ganglion. Certain voltage-gated sodium channels, such as Na_v_ 1.8, are necessary for action potential firing and sensing pain. Prior work has demonstrated that interfering with voltage-gated calcium channel activity can also reduce acute and chronic pain. Furthermore, it is reported that dorsal root ganglion calcium response to electric-field stimulation was inhibited by voltage-gated sodium channel blockers. Here we aim to explore the intracellular calcium dynamics in dorsal root ganglion neurons in response to cold pain via intravital confocal imaging. Genetically encoded calcium indicators, such as the GCaMP family of proteins, have been widely used to assess neuron excitability, using fluorescence due to calcium flux as a surrogate for neuron depolarization. We utilized Thy1-GCaMP6f transgenic mice to image calcium transients in live animals or freshly-isolated dorsal root ganglion neurons to identify neuron activity. Measurement of calcium transients demonstrated response to a TRPV1 agonist, capsaicin, or a voltage-gated sodium channel agonist, veratridine, and this response could be inhibited by capsazepine: a TRPV1 antagonist; carbamazepine: a pan-voltage gated sodium channel blocker; or A-803467: a Na_v_ 1.8 inhibitor. We demonstrated through intravital imaging that the cold paw swab increases calcium transients in dorsal root ganglion neurons, and this effect was inhibited by voltage-gated calcium channel blockers. We further demonstrated by *in vivo* imaging that the cold-pain induced calcium transients in dorsal root ganglion neurons is blocked by A-803467. These data demonstrate that imaging can be used to visualize and measure cold pain signaling in dorsal root ganglion neurons due to L-type voltage-gated calcium channel activity triggered by Na_v_ 1.8 activation.

## Introduction

Primary sensory neurons of the peripheral nervous system carry various sensations from the extremities to the central nervous system [1,2]. The cell bodies of these sensory neurons are organized in ganglia, proximal to the spinal column. Along with support cells (e.g., satellite glial cells) and blood vessels, this complex microenvironment constitutes the dorsal root ganglion (DRG). The DRG is the first to process external stimuli, including pain and cold sensation [3]. Chronic pain conditions are a result of unwanted and uncontrolled depolarization of DRG neurons. Among chronic pain sufferers, cold hypersensitivity, or cold allodynia, is a major occurrence [3,4]. Patients that suffer from this condition experience noxious cold pain at a significantly warmer threshold. Most people experience 20°C temperatures as unpleasant, but not painful [4,5]. However cold allodynia sufferers experience a noticeable pain sensation below 20°C. This hypersensitivity increases exponentially as the temperature drops, down to 5°C, beyond which tissue damage begins [4,6]. Pharmacological targeting of DRG neurons, with the goal of reducing aberrant neuron depolarization, is a promising avenue of treatment for chronic pain patients.

Action potential generation leads to membrane depolarization, which causes calcium influx through voltage gated calcium channels (VGCCs) [7–11]. The intracellular calcium triggers neurotransmitter release at the presynaptic terminal thus passing the signal along through the network of neurons to the spinal cord and central nervous system [7]. VGCCs are activated by high-frequency membrane depolarization and are often a target of pain intervention [12,13]. L-type VGCCs have slow voltage-dependent inactivation, which leads to long-lasting Ca^2+^ influx. Calcium entry via other membrane receptors/channels also trigger pain sensations, such as the TRPV1 channel, which is activated by several factors including capsaicin [14]. Conversely, voltage-gated sodium channels (VGSCs) are responsible for priming neuron depolarization by permitting initial sodium ion influx and beginning the change in membrane potential needed for depolarization [15–17]. One of the most highly expressed voltage gated sodium channels (VGSC) in the DRG nociceptive neurons is Na_v_ 1.8 [18]. It is responsible for cold-induced nociception in small sensory neurons of the DRG [18–20], although there is some conflicting data for the role of Na_v_ 1.8 expression specifically on cold-sensing DRG neurons [21]. Studies have demonstrated that as the temperature lowers, Na_v_ 1.8 channels reduce their activation threshold, resulting in an increase of cold sensitivity [20].

Na_v_ 1.8 small molecule inhibitors and KO mouse models have shown that Na_v_ 1.8 is responsible for transmitting cold pain stimulus [19,20,22,23]. Previous studies have used calcium imaging to monitor functional activity of pain-sensing neurons *in vivo* [24,25]. However, there is sparse direct *in vivo* data to understand the complex mechanisms underlying DRG calcium transients in cold pain sensing functions. In this work we utilize well-established methods for modulating cold pain response VGSCs and VGCCs to demonstrate direct in vivo visualization and quantification of DRG neuron activity. We used the Thy1-GCaMP6f [26] transgenic mouse to visualize calcium entry in the DRG neurons both *in vivo* and *ex vivo*. Prior studies have demonstrated the effectiveness of using GCaMP signaling to measure Na_v_ 1.8-related neuron activation [27]. Our data utilizes GCaMP imaging to directly visualize and quantify the critical function of VGSCs to trigger calcium influx.

## Materials and methods

### Chemicals

The following chemicals were used: veratridine at 100 µM (MilliporeSigma: 6769505MG), capsaicin at 1 µM (Sigma Aldrich: 501656999), calcium ionophore at 10 µM (Sigma Aldrich: A23187), carbamazepine at 5 µM (TocrisBiosciences: 40-985-0), capsazipine at 10 µM (TocrisBiosciences: 046410), cadmium chloride at 200 µM (Thermo Scientific Chemicals: AC219141000), Nifedipine at 200 µM (Tocris Biosciences: 1075), A-803467 at 10 µM (Thermo Scientific Chemicals: AAJ67281MA).

### Experimental animals

Homozygous Thy1-GCaMP6f mice (GP5.17) [26] at least 8 weeks old at the time of experiment were used for all experiments. The mouse *Thy1* promoter drives expression of GCaMP6f in multiple types of neurons, including a subset of sensory neurons in the DRG. GCaMP6f is a genetically encoded calcium indicator. All animal procedures were performed in accordance with protocols approved by the Institutional Animal Care and Use Committee (IACUC) of Eli Lilly and Company.

### DRG calcium imaging

DRG calcium imaging was performed on the Zeiss LSM 980 confocal microscope and 10x air objective (NA = 0.45, Carl Zeiss). Samples were excited using the 488 nm laser and images were collected at ~2 Hz, with an image size of 512x512 pixels for a time lapse of 2 minutes. For *in vivo* images, an optical inverter (LSM Tech) was used to image the DRG from the dorsal side of the animal while it was secured ventral-side down.

#### *Ex vivo* calcium imaging.

To extract DRG for *ex vivo* imaging, Thy1-GCaMP6f mice were placed under 3% isoflurane anaesthesia then sacrificed via cervical dislocation. Lumbar DRGs were isolated and put in Neurobasal™-A Medium, minus phenol red (ThermoFisher:12349–015). They were then placed on glass coverslips and secured with small glass pieces and liquid surface tension such that they were held motionless during imaging but still exposed to treatments, as necessary. Samples were kept at 37° C during imaging.

#### *In vivo* calcium imaging.

Thy1-GCaMP6f mice were anesthetized using 3% isoflurane and kept on a heated surgical surface to maintain body temperature. Terramycin was applied to keep eyes from drying out and the back was shaved to expose skin. The last rib, T13, was located by touch because the L4 DRG is located where it joins the vertebral column. The skin was cut to expose the spine on the dorsal surface. Then the muscle covering the vertebral surface was removed. The L4 or L5 DRG was visually located on the left side and the spinous process was cut away. A small hole was made on the lamina using a hand-held dental drill. Rongeurs were used to cut away the left dorsal surface of the vertebrae corresponding to the L4 or L5 DRG and the DRG was carefully exposed by cutting away the bone over the DRG. Following surgery, isoflurane was reduced to 1.5% in oxygen to maintain surgical plane of anesthesia while reducing neurological suppression. Animal was placed on the stage of LSM980 confocal microscope where the microscope environmental chamber was kept at 33°C. The ipsilateral hind paw was stimulated by gently swabbing the paw with a cotton-tipped applicator dipped in ice cold 100% acetone [28]. Nifedipine at 20 mg/kg [29,30] or A-803467 [22,31] at 70 mg/kg were administered via intraperitoneal injection (i.p.) prior to the surgical DRG preparation under isoflurane anesthesia.

### Image data and statistical analysis

Images were analysed using Fiji [32]. Regions of interest (ROI) corresponding to responding somas were identified visually and circles smaller than the somas were drawn around each. For *ex vivo* image analysis, we measured baseline activity in an average of 5 ROIs per DRG before treatment then measured neuron activity 1 minute post-stimulation. For *in vivo* image analysis, we measured average baseline and response from at least 3 neurons per DRG. Baseline activity was measured from images for 30 seconds before stimulus was applied. The peak of fluorescence after stimulus was used as the maximal response for each soma. To correct for autofluorescence signal, we performed a background subtraction based on a rolling ball average of the non-DRG pixels in the image. ∆F/F was calculated as calcium transit: ∆F = F_t_ -F, where F_t_ is fluorescence intensity at the time point of interest, and F is the baseline intensity. Results are expressed as the mean ± SEM. Ordinary one-way ANOVA was used to generate the selective pairwise comparisons for *ex vivo* DRG images. Paired t-test was used to compare between treatment groups captured by *in vivo* imaging. Analyses were performed using GraphPad Prism 9.5.0, using p < 0.05 as the cutoff for statistical significance.

## Results

### Imaging of GCaMP6f provides measurement of neuron activity

**Fig 1:**
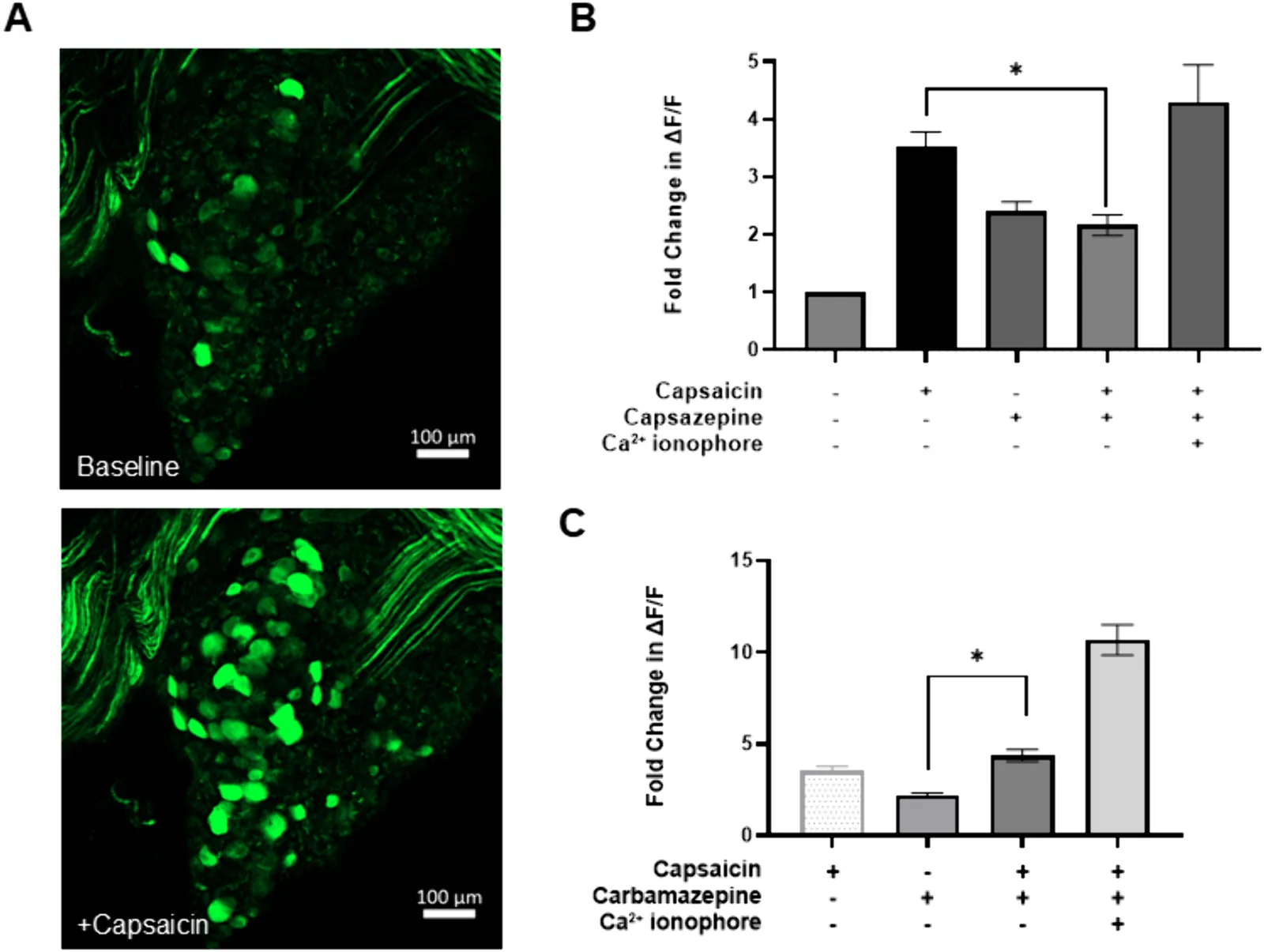
Capsaicin induced Ca2 + influx in freshly isolated DRG. Ex vivo DRG isolated from Thy1-GCaMP6f mice (N = 4 mice per treatment) were imaged before and after exposure to capsaicin, with capsazepine or carbamazepine pre-treatment for 10 or 5 min, respectively. Ca2 + ionophore was applied to the DRG at the end of experiments. (A) Representative images of baseline ex vivo DRG neurons and response after stimulation. The fold change in ∆F/F is compared between baseline to 1 min after capsaicin with or without treatment of (B) capsazepine or (C) carbamazepine. To conserve animals for this study, control capsaicin-only data (n = 4 mice) in (C) is reused control data from (B). All bar graphs represent the mean ± SEM. Ordinary one-way ANOVA was used to generate selective pairwise comparisons. * p < 0.05.

To validate the functional readout of DRG in the Thy1-GCaMP6f transgenic mice, we tested capsaicin which is known to cause calcium influx via TRPV1 as well as neuron depolarization. Capsaicin treatment in freshly isolated DRGs from Thy1-GCaMP6f mice significantly increased ∆F/F compared to untreated control (Fig 1A). Pretreatment with the TRPV1 antagonist, capsazepine, significantly reduced the ∆F/F response to capsaicin (Fig 1B), confirming the ∆F/F response was mediated by TRPV1-induced calcium influx. Following each ex vivo imaging experiment, we added a calcium ionophore to force calcium influx in the neurons via chemically-induced membrane pores. This was done to confirm DRG neurons were alive and capable of a GCaMP response. To test the contribution of VGSG in capsaicin-evoked Ca^2+^ responses, we measured the effect of a VGSC blocker, carbamazepine, on the capsaicin-evoked responses. These responses were not inhibited by carbamazepine (Fig 1C), suggesting that the Ca^2+^ response is mediated by the calcium influx via TRPV1 channel per se, rather than VGSC-mediated depolarization.

### VGSC-mediated sodium influx is necessary for calcium transients

There are many voltage-gated ion channels that are necessary for neuron depolarization. However, we posit that one specific VGSC, Na_v_ 1.8, is required for response to noxious cold stimuli. The first question was whether use of imaging could delineate the specific activation of neurons via VGSCs. To interrogate the interplay between voltage-gated sodium and calcium ion channels, we utilized several different types of agonists and antagonists in an *ex vivo* imaging configuration.

**Fig 2:**
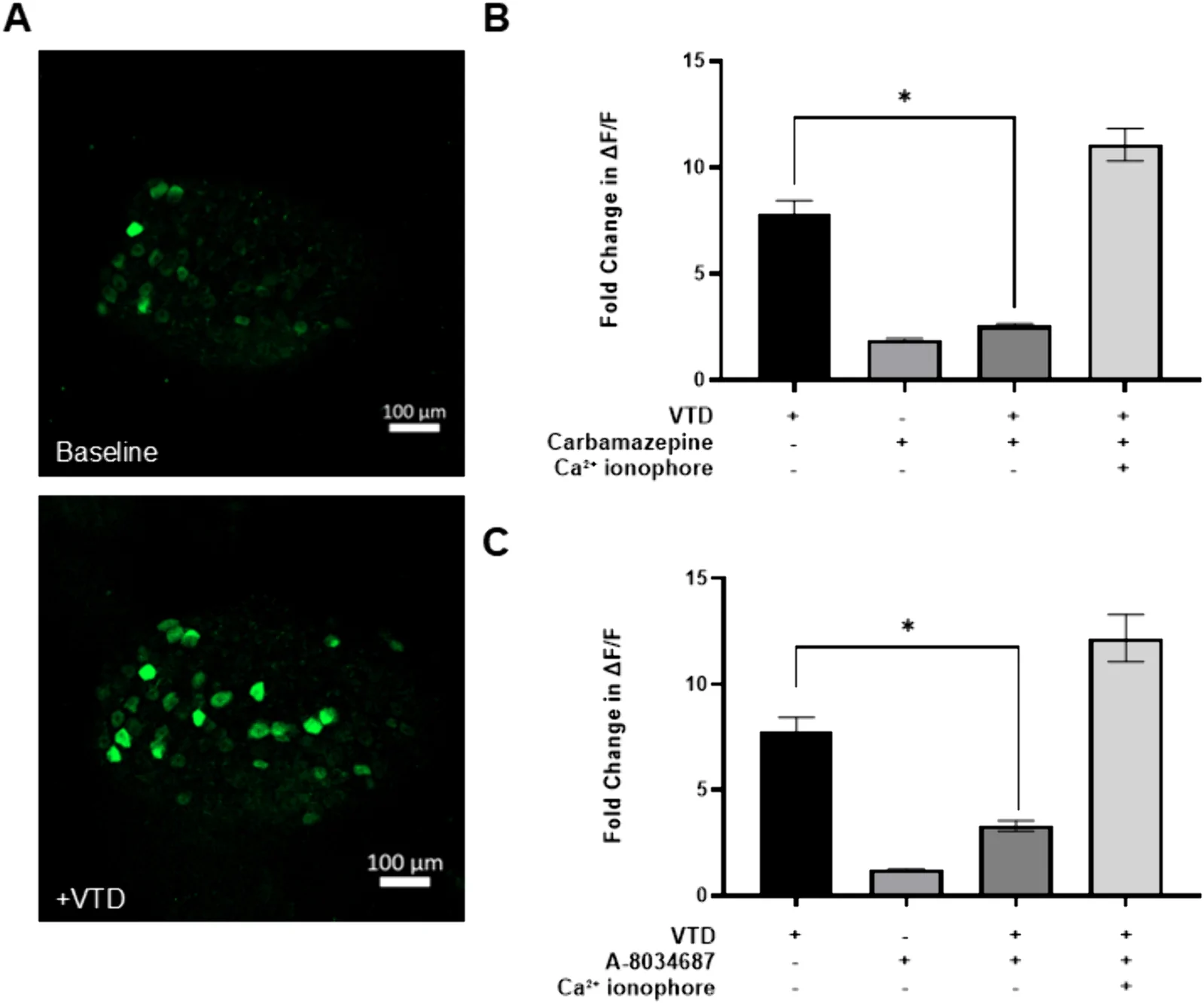
Veratridine induced Ca2 + influx in freshly isolated DRG. Ex vivo DRG isolated from Thy1-GCaMP6f mice (N = 4 mice) were imaged before and after exposure to veratridine (VTD), and with carbamazepine or A-8034687 pretreatment for 5 or 2 min, respectively. Ca2 + ionophore was applied to the DRG at the end of experiments. (A) Representative images of DRG calcium responses. The fold change in baseline ∆F/F compared 1 min after VTD with or without treatment of (B) carbamazepine or (C) A-8034687. To conserve animals, ∆F/F data from DRG treated with VTD only (positive control) is used for (C) and (B). All bar graphs represent the mean ± SEM. Ordinary one-way ANOVA was used to generate the selective pairwise comparisons. * p < 0.05.

To test the role of VGSCs on Ca^2+^ influx, we treated DRGs *ex vivo* with Veratridine (VTD), which is a known VGSC agonist that cause VGSCs to stay open by inhibiting channel inactivation [33]. Incubation in VTD is expected to increase Na^+^ concentration in the DRG neuron, which leads to depolarization [33–35]. We incubated the *ex vivo* DRGs in VTD for 1 minute and measured a significant increase of Ca^2+^ response in ∆F/F compared to baseline (Fig 2A and 2B). However there was some consideration to whether the *ex vivo* neurons were responding to any stimulation or if this response was specific to VGSCs. The sodium channel blocker, carbamazepine, binds to the inactive form of VGSCs and inhibits the effect of VTD on neurons. We incubated the DRGs in carbamazepine alone to verify that it minimally affects basal calcium levels (Fig 2B). We then pre-treated the neurons with carbamazepine for 5 minutes, followed by application of VTD. In Fig 2B we showed that carbamazepine significantly inhibited DRG calcium influx induced by VTD, confirming that sodium influx via VGSCs is required for calcium influx in DRG neurons. To specifically interrogate Na_v_ 1.8 function, we treated *ex vivo* DRGs with A-803467, a Na_v_ 1.8 inhibitor [22,36]. A-803467 has been established as a potent and selective inhibitor of Na_v_ 1.8. Measured IC50 values show multi-fold difference in blocking efficacy when comparing Na_v_ 1.8 (0.079 uM) to other Nav channel subtypes, such as Na_v_ 1.7 (35.34 uM) or Na_v_ 1.9 (activity too low to be measured) [22]. Given this large disparity in IC50 levels and the evidence that demonstrates Na_v_ 1.8 is the predominant Na_v_ family channel expressed in mouse DRG, we assume the GCaMP response measured via confocal imaging is almost entirely from Na_v_ 1.8 activity [37]. A-803467 treatment significantly reduced the measured calcium influx in *ex vivo* DRGs (Fig 2C). However, the Ca^2+^ response to VTD was not completely blocked, suggesting that VTD activates VGSCs indiscriminately. Therefore these data demonstrate that sodium influx via Na_v_ 1.8 is a critical for Ca^2+^ influx in DRG neuron.

### L-type VGCC are responsible for the VTD-induced calcium influx

**Fig 3:**
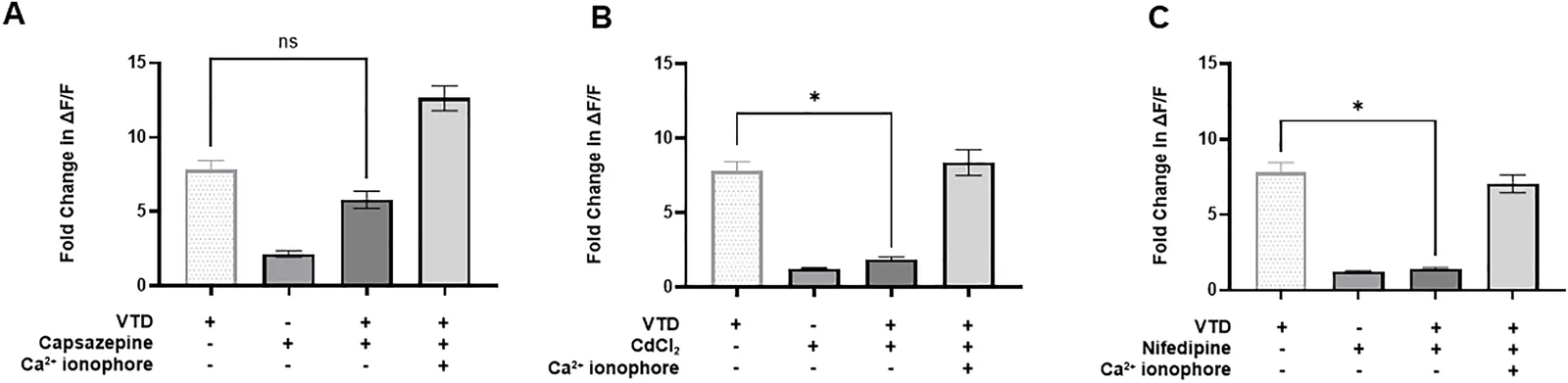
Effects of calcium channel inhibitors on veratridine-induced Ca2 + influx in freshly isolated DRG. Ex vivo DRG isolated from Thy1-GCaMP6f mice (N = 4) was imaged before and after exposure to veratridine (VTD), and with CdCl2, Nifedipine, or capsazepine pretreatment. Ca2 + ionophore was applied to the DRG at the end of experiments. The fold change in ∆F/F compared to baseline at 1 min after VTD with or without treatment of (A) Capsazepine, (B) CdCl2, or (C) Nifedipine. To conserve animals, ∆F/F data from DRG treated with VTD only (positive control) is used for (B) and (C), was generated from experiments in Fig 2B. All bar graphs represent the mean ± SEM. Ordinary one-way ANOVA was used to generate the selective pairwise comparisons. * p < 0.05. ns, not significant.

The relationship between VGSCs and neuron depolarization is complex. While prior publications have shown the necessity for functioning VGSCs, and specifically Na_v_ 1.8, in depolarization, many downstream signal cascades following an action potential require an influx of extracellular calcium through VGCCs [15,38,39]. We demonstrated that use of carbamazepine to sustain inactivation of VGSCs was insufficient to block downstream calcium influx (Fig 1C). Furthermore, VTD-induced activation of VGSCs and the downstream calcium influx was not inhibited by specific inhibition of TRPV1 through capsazepine (Fig 3A). Use of a non-specific VGCC inhibitor, cadmium chloride (CdCl_2_), significantly reduced calcium influx induced by VTD treatment (Fig 3B). To specify the VGCC classes, we tested Nifedipine, a well-known blocker of L-type VGCCs. Pre-treatment of Nifedipine inhibited VTD-induced calcium influx in the *ex vivo* model (Fig 3C). These data demonstrate that L-type VGCCs mediate calcium influx triggered by VGSC activation as detected by somatic GCaMP signals in DRG neurons.

### *In vivo* inhibition of L-type VGCCs reduce response to cold pain stimulus

**Fig 4:**
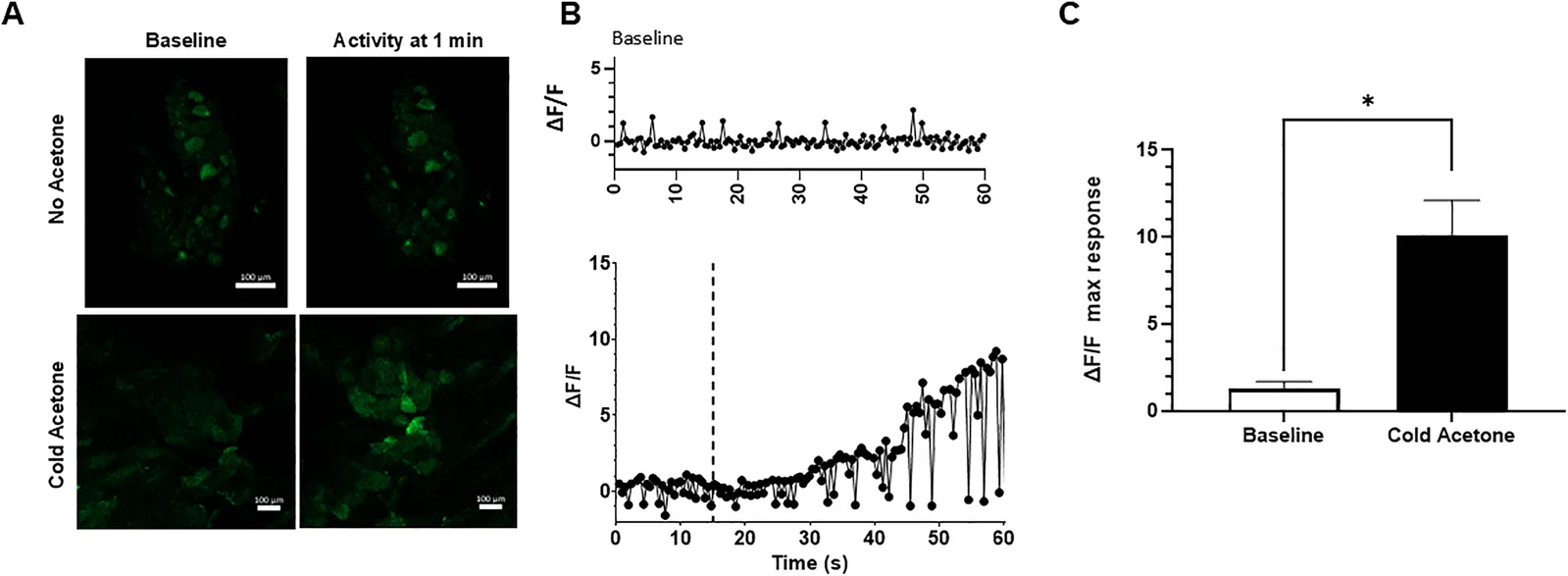
Intravital imaging of DRG to observe Ca2 + influx in response to cold pain stimulus. Under anesthesia, L4/L5 DRG in Thy1-GCaMP6f mice (N = 5) was surgically exposed and imaged. Cold acetone was applied to ipsilateral hind paw via swab at t = 15 s (indicated by the dotted line). (A) Representative images of DRG calcium responses, and arrows indicate GCaMP6f expressing neurons which show spontaneous activity (control), and responses to cold acetone after observation for at least 1 minute. Scale bar 25 µm. (B) Representative trace of ∆F/F over time for spontaneous baseline activity (above) as well as response to cold acetone (below). (C) Bar graphs showing average ∆F/F of spontaneous baseline activity over 30s and the maximum peak response of DRG neuron responding to cold acetone swab. Bar graphs represent the mean ± SEM and paired t-test was used for analysis. * p < 0.05.

Our central question is whether VGSCs, like Na_v_ 1.8, are critical for sensing noxious cold stimuli and whether this inhibition can be captured using *in vivo* imaging. *Ex vivo* experiments cannot sufficiently replicate the noxious cold sensing that occurs *in vivo*. In addition, the *in vivo* response to cold pain has not been previously captured via imaging of the Thy1-GCaMP6f mouse model. We established that imaging in the Thy1-GCaMP6f mice was sufficiently sensitive to detect responses to cold pain stimulation (Fig 4). By soaking a cotton-tipped applicator in chilled 100% acetone, we gently and briefly swabbed the paw pad to induce cold pain. When the mice were swabbed with cold acetone, a significant increase in GCaMP6f signal and ∆F/F was measured compared to spontaneous neuron activation in the control animal (Fig 4A), thereby assuming an activation of the pain response (Fig 4A, B). In a representative field of view, we saw an average of 3 DRG neurons respond to cold stimulus, out of approximately 17 neurons visible (Fig 4A). In Fig 4B, we show representative ∆F/F traces for baseline activity followed by DRG neuron response after cold acetone swab. Dips in the *in vivo* traces are due to animal breathing while under anaesthesia and are disregarded for downstream calculations. When we compare the average maximum ∆F/F from multiple animals, cold pain swab demonstrates significantly higher signal compared to the baseline, showing the efficacy of measuring cold response via imaging (Fig 4C).

**Fig 5:**
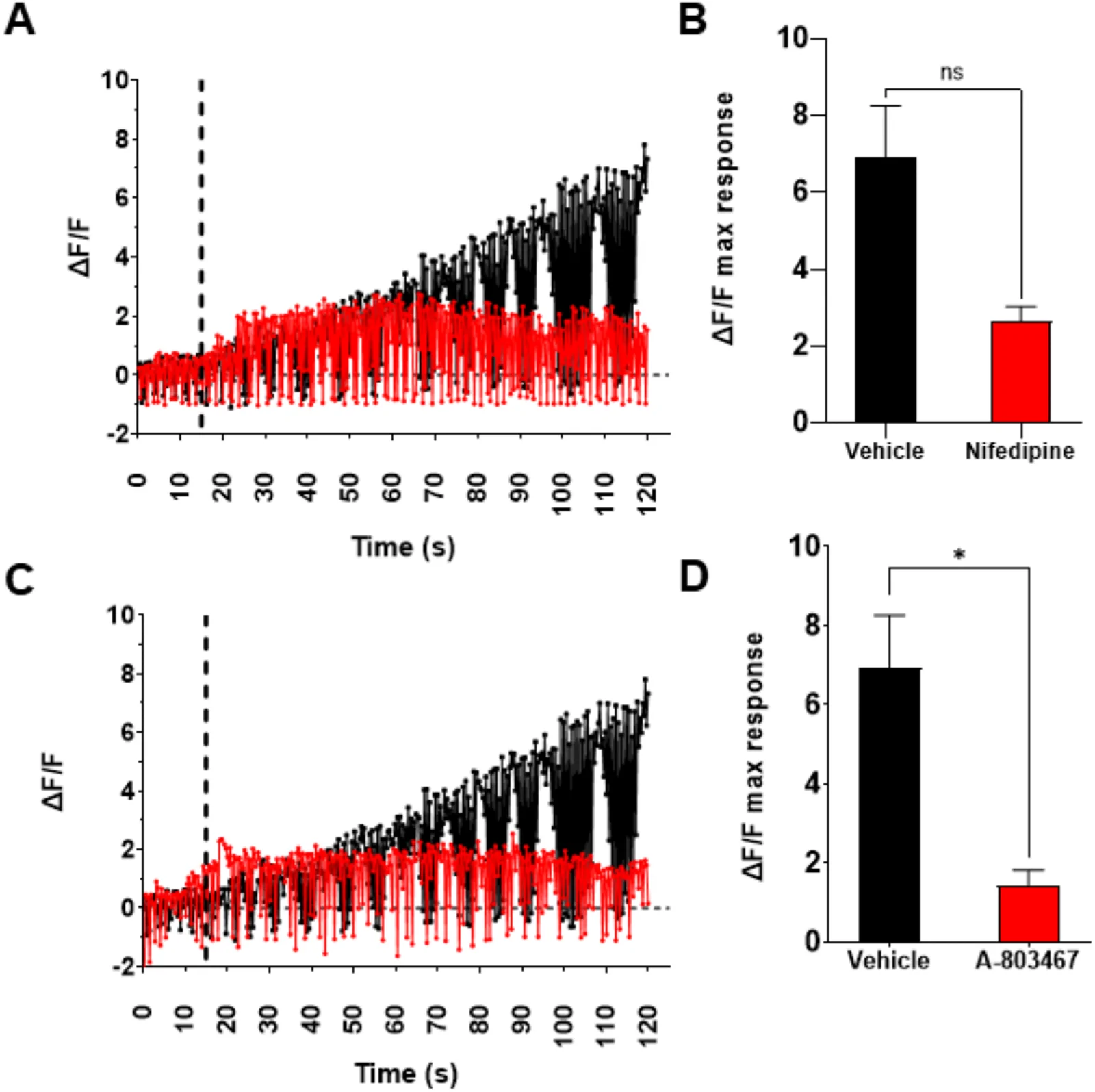
In vivo inhibition of ion channels during noxious cold stimulation. Under the anaesthesia, L4/L5 DRG in Thy1-GCaMP6f mice (N = 4 per treatment group) was surgically exposed and imaged. Cold acetone was applied to ipsilateral hind paw at 15 sec after image collection started (dotted line). (A, B) Nifedipine was administered i.p. at 20 mg/kg 30 min before mice were stimulated with cold acetone paw swab. (A) Representative trace of ∆F/F of vehicle treatment group (red line) compared to Nifedipine treatment group (black line). (B) Bar graphs showing average ∆F/F of the maximum peak response of DRG neuron responding to cold acetone swab. Bar graphs represent the mean ± SEM and unpaired t-test was used for analysis. ns, not significant. (C, D) A-803467 was administered i.p. at 70 mg/kg 30 min before mice were stimulated with cold acetone paw swab. To conserve mice, vehicle treatment group was reused from (A, B). (C) Representative trace of ∆F/F over time for vehicle treatment (red line) compared to A-803467 treatment (black line) in mice stimulated with cold acetone paw swab. (D) Bar graphs showing average ∆F/F of the maximum peak response of DRG neuron responding to cold acetone swab. Bar graphs represent the mean ± SEM and unpaired t-test was used for analysis. * p < 0.05.

We next investigated the involvement of VGCCs in cold pain-induced DRG calcium transients. We dosed nifedipine at 20 mg/kg via i.p. injection in Thy1-GCaMP6f mice and then measured DRG neuron responses following cold acetone swab. We observed the reduction of ∆F/F (Fig 5A-B) compared to un-treated cold pain-stimulated animals, but the effect was not strong enough to be statistically significant. This suggests that downstream signalling of L-type VGCCs are involved in the *in vivo* response to cold pain stimuli, but more data is needed in this experimental model to fully elucidate this relationship. Next, to specifically test the involvement of Nav 1.8 *in vivo,* A-803467 was administered via i.p. injection at 70 mg/kg 30 minutes prior to hind paw cold stimulation [22]. A-803467 significantly inhibited DRG neuron ∆F/F, with the vehicle control showing minimal inhibitory effect (Fig 5C-D). These data demonstrate that both Na_v_ 1.8 and VGCC play a role in the signalling response to cold-induced pain stimuli.

## Discussion

Neurons within the dorsal root ganglion are in an optimal physiological location for pharmacodynamic targeting of pain-signal transmission. However, targeting and inhibiting signaling mechanisms in pathological conditions, like chronic pain, is not simple. Complete blockage of pain signaling carries dangerous implications, as we know from patients suffering from congenital insensitivity to pain (CIP) [40]. Effective treatment requires precise targeting of specific pain signal propagation, either by targeting action potential propagation or preventing depolarization of specific neuron subtypes. One important step in developing novel therapeutics is understanding the interplay between the different ion channels *in vivo*. Cold allodynia is a common symptom in patients suffering from chronic pain and is a consistent and reproducible noxious stimulus we can use to evaluate neuron activity. It is reported that TRPM8 null mice showed a significant reduction in injury-induced behavioral responsiveness to acetone cooling, suggesting that calcium signaling is involved in cold pain sensation [41]. In our study, we directly monitor calcium dynamics *in vivo* in DRG neurons using confocal imaging and the Thy1-GCaMP6f transgenic mouse. We validated the functional output of this assay by stimulating the ipsilateral hind paw with a cold acetone swab to stimulate pain sensations. We measured the change in GCaMP fluorescence and confirmed this flux was coincident with paw pad stimulation.

Many investigations into pain sensing in the DRG have revealed potential ion channel targets that can strike the balance as being necessary for pain sensing but not globally expressed throughout the nervous system. One such target is the VGSC, Na_v_ 1.8. Clinical trial data support the blockage of Na_v_ 1.8 as a reasonably safe avenue for attenuating pain, with low expression of Na_v_ 1.8 in the CNS de-risking adverse events due to brain-specific inhibition [42]. However, Na_v_ 1.8 expression in cardiac myocytes hints at a more complicated function in the heart [43,44]. While there are conflicting reports on whether Nav 1.8-expressing DRG neurons directly sense cold-pain [21,45], sufficient data in the literature support that small-molecule antagonism of Na_v_ 1.8 significantly inhibits neuron response. We first sought to better understand how VGCCs and VGSCs are involved DRG neuron pain signaling by measuring calcium influx that occurs following neuron depolarization. Due to limited number of approved *in vivo* VGSC inhibitors, we investigated mechanisms underlying DRG calcium dynamics using *ex vivo* DRG. After inhibiting activation of VGSCs via carbamazepine, we measured a reduction in calcium ΔF/F response, confirming that calcium entry is downstream of neuron excitation. Broad inhibition of VGCCs with CdCl_2_ also demonstrated a reduction in calcium influx, even after VGSC stimulation, suggesting that VGCC activation occurs following membrane depolarization in DRG. Through these experiments we also noted that singular inhibition of calcium-permeable afferent receptors, such as TRPV1, was not sufficient to prevent neuronal excitation through VGSC stimulation with VTD, which acts on VGSCs downstream of TRPV1. It is reported that TRPM8 and TRPA1, nociceptor transduction ion channels with cool or cold activation temperatures, showed reduced expression in a chronic constriction injury model of neuropathic pain in mice but this expression loss did not alter calcium response to cold stimuli in *ex vivo* DRG [46]. These data suggest that in cases of inhibited or reduced expression of calcium-permeable receptors, stimulation of VGSCs can still elicit neuron responses. It is possible that transient receptor potential superfamily of nociceptor transduction ion channels may not have a direct role in DRG soma. It should also be noted that intercellular stores of calcium (largely in the ER) could also contribute to the observed level of calcium response during stimulation. It has demonstrated that neurons may rely on these calcium stores to accentuate ion release when a weak stimulus is detected [47]. Our work was not designed to elucidate intracellular versus extracellular calcium stores, however even intracellular release is predicated by external stimuli. Therefore any contribution of intracellular calcium to the GCaMP signal can still be classified as a response to noxious cold. This complex biology requires further study to investigate DRG soma calcium influx in response to peripheral activation of TRPs.

Na_v_ 1.8 is an attractive target for inhibiting noxious cold pain transmission to the spinal cord because it is expressed in almost all small-diameter nociceptive neurons and over 40% of large diameter neurons of the DRG [14,48]. *In vivo* recordings from the spinal dorsal horn show that Na_v_ 1.8 is necessary for sensing noxious cold stimuli and ablation of this ion channel significantly reduces neuron activity upon noxious cold stimulus [39,49]. Timed inhibition of Na_v_ 1.8 has shown to reduce neuropathic pain in rats [39]. These data demonstrate the critical role for Na_v_ 1.8 in sensing pain. There are even small molecule inhibitors specific to Na_v_ 1.8, such as A-803467, that have shown in vitro efficacy of blocking action potentials [22]. A-803467 has even been shown effective in reducing behavioral end points of chronic pain in rats [31]. However, one challenge with small molecule inhibitors can be off-target inhibition of family-member proteins. In the case of Na_v_ 1.8, there are multiple Na_v_ family members that play critical roles in peripheral neuron depolarization and sympathetic neuron depolarization. Even within DRG neurons, unintended modulation of Nav 1.7 or Na_v_ 1.9 can cause dramatic changes in peripheral sensing. Prior studies have demonstrated that A-803467 has high potency and specificity for Nav 1.8 [22]. While it has some inhibition effect on Na_v_ family members (e.g., IC_50_ of 35.34 uM on Na_v_ 1.7), the potency for Na_v_ 1.8 is orders of magnitude higher (e.g., IC_50_ of 0.079 uM); therefore, we presume that *in vivo* dosing of A-803467 has minimal effect on non- Na_v_ 1.8 proteins.

In addition to Nifedipine, we observed that A-803467 inhibits calcium response to VTD in *ex vivo* DRGs. However no experiments have been done directly measuring the *in vivo* functional relationship of Na_v_ 1.8 inhibition to calcium influx. We tested the DRG neuron response to a large-scale blockage of L-type VGCCs by dosing Nifedipine before stimulating the paw pad with a cold acetone swab. The observed trend suggested that L-type VGCC blockage does reduce calcium influx even with neuron stimulation. Importantly, administration of A-803467 showed a statistically significant inhibition of DRG calcium influx compared to vehicle controls following noxious cold paw pad stimulation. This behavior was reproduced across multiple animals and confirms our hypothesis that Na_v_ 1.8 function is necessary for downstream calcium-related signaling after cold pain stimulation. Blockage of Na_v_ 1.8 causes a reduction in calcium influx, which could have dramatic effects on pain signaling pathways *in vivo*. Given the specificity of A-803467, this supports the role of Na_v_ 1.8 over Na_v_ 1.7 or Na_v_ 1.9 for noxious cold sensing. Our novel data also demonstrate the utility of leveraging *in vivo* imaging to capture the calcium response of Na_v_ 1.8-expressing neurons in cold nociception and signal transduction. Leveraging this technology for DRG neuron activity could accelerate development of therapies targeting peripheral pain disorders. However additional caution should be exercised when targeting Nav 1.8 for pain abrogation in humans as its role in cardiac tissue could create complications during treatment. And while clinical data has demonstrated the safety of inhibiting Nav 1.8, the effect on other peripheral nociception that occurs in the DRG neurons should be monitored for unexpected alterations to signaling pathways.

## Supporting information

S1 Fig Under anesthesia, L4/L5 DRG in Thy1-GCaMP6f mice (N = 1) was surgically exposed and imaged. Room temperature acetone on a sterile swab was applied to ipsilateral hind paw at the time point indicated by the dotted line (15 sec). ΔF/F response from 5 neurons in the DRG are plotted over the course of 60 seconds of imaging.
(TIF)

S1 Raw Data Raw data, following initial processing from instrument, used to quantitate response traces in Fig 1-5. (XLSX)
